# Detection of asinine gammaherpesviruses in association with pulmonary fibrosis in free-ranging donkeys

**DOI:** 10.1177/10406387211052998

**Published:** 2021-10-23

**Authors:** Grazieli Maboni, E. Jane Kelly, Chad S. Clancy, Eliana De Luca, Thomas J. Baldwin, Arnaud J. Van Wettere, Albert J. Kane, Summer Peterson, V. Gus Warr, Dona A. Bastian, Susan Sanchez

**Affiliations:** Department of Pathobiology, Ontario Veterinary College, Guelph, Ontario, Canada; Athens Veterinary Diagnostic Laboratory, University of Georgia, Athens, GA, USA; Utah Veterinary Diagnostic Laboratory, Logan, UT, USA; Utah Veterinary Diagnostic Laboratory, Logan, UT, USA; Athens Veterinary Diagnostic Laboratory, University of Georgia, Athens, GA, USA; Utah Veterinary Diagnostic Laboratory, Logan, UT, USA; Utah Veterinary Diagnostic Laboratory, Logan, UT, USA; USDA-APHIS Veterinary Services, Fort Collins, CO, USA; Spring City, UT, USA; Utah Wild Horse and Burro Program, Bureau of Land Management, Salt Lake City, UT, USA; Utah Wild Horse and Burro Program, Bureau of Land Management, Salt Lake City, UT, USA; Athens Veterinary Diagnostic Laboratory, University of Georgia, Athens, GA, USA

**Keywords:** burros, donkeys, gammaherpesviruses, mortality, pneumonia, pulmonary fibrosis

## Abstract

A mortality event among recently captured feral donkeys (*Equus asinus*) occurred in south-central Utah in 2016. The deaths were sporadic, and clinical signs were indicative of respiratory disease, likely associated with an infectious etiology. Ten of 13 donkeys autopsied had moderate-to-severe interstitial fibrosing pneumonia, and one had pyogranulomatous pneumonia. Consensus PCRs directed toward the DNA polymerase and DNA packaging terminase subunit 1 for herpesviruses were performed followed by sequencing of the PCR amplicons and phylogenetic analysis. Asinine herpesvirus 4 (AsHV4) and 5 (AsHV5) were consistently identified in lung tissues of affected donkeys. No other herpesviruses were identified, and herpesviral DNA was not detected in lung tissues of 2 donkeys without evidence of respiratory disease. The detection of asinine gammaherpesviruses may have been associated with the lesions described. AsHV4 and AsHV5 have been reported in previous studies as novel gammaherpesviruses based on sequences obtained from donkeys with interstitial pneumonia and marked syncytial cell formation. Our findings suggest that the association of asinine gammaherpesviruses with respiratory conditions in equids deserves further attention.

Herpesviruses are important pathogens associated with respiratory disease in equids.^10^ The *Herpesviridae* family comprises viruses that have been grouped into the subfamilies *Alpha*-, *Beta*-, and *Gammaherpesvirinae* according to their biologic and genomic features. Gammaherpesviruses have been associated with several respiratory conditions including pulmonary fibrosis in equids, humans, and rodents.^10,19^ Equid gammaherpesvirus 5 (EHV5) has been implicated particularly in equine multinodular pulmonary fibrosis, which is a chronic progressive disease characterized by clearly demarcated nodules of fibrosis in the lung.^19,20,22^

Little information is available about asinine gammaherpesviruses. A few asinine gammaherpesviruses have been associated with various clinical conditions in equids.^2,3,7,9,10^ In particular, asinine herpesvirus 4 (AsHV4) and 5 (AsHV5) were designated as novel gammaherpesviruses based on sequences obtained from donkeys with interstitial pneumonia and marked syncytial cell formation.^7^ Donkeys infected with AsHV4 and AsHV5 were reported to develop acute and often fatal respiratory disease.^7^ Besides donkeys, AsHV5 was described in horses with pyogranulomatous pneumonia,^4^ and with multinodular pulmonary fibrosis.^1^ Further, the pathogenic potential of asinine gammaherpesviruses was reinforced by its association with neurologic disease in a donkey.^18^ However, given that asinine gammaherpesviruses have also been detected from clinically healthy equids,^11,13^ their pathogenic role has not yet been fully elucidated.

Here, we describe a cluster of respiratory cases associated with a mortality event in feral donkeys (*Equus asinus*) from which pulmonary lesions were characterized by fibrosing pneumonia with consistent detection of AsHV4 and AsHV5. To our knowledge, asinine gammaherpesviruses have not been described in association with an outbreak condition in either domestic or feral donkey populations.

In April 2016, 236 feral donkeys were gathered in south-central Utah, of which 133 were retained and 103 were returned to the range as part of a planned herd reduction. Mortality was higher than expected among the recently captured animals. Deaths were sporadic, and clinical signs were indicative of respiratory disease, likely associated with an infectious etiology of relatively slow spread. No other epidemiologic data was available. Between April and July, 13 of the 26 animals that died were submitted for postmortem examination at the Utah Veterinary Diagnostic Laboratory (Logan, UT, USA). Body condition scores were 3–4 (on a Henneke scale of 1–9), and the donkeys were 1–30 y old (Table 1). Histologic lesions in 10 of 13 donkeys autopsied included moderate-to-severe, fibrosing interstitial pneumonia with moderate numbers of macrophages and fewer neutrophils and/or proteinaceous fluid in adjacent alveolar spaces (Figs. 1, 2). Pyogranulomatous pneumonia was diagnosed in one animal. Five donkeys had regions of myxomatous stroma expansion of alveolar septa (Fig. 2). Frequently, regions of alveolar septal expansion compressed the adjacent alveolar spaces and entrapped small numbers of foamy macrophages. Rare multinucleate cells were observed within terminal airways and alveolar spaces (Fig. 2), and rare alveoli were lined by hyaline membranes. Viral inclusion bodies were not identified. Unlike equine multinodular pulmonary fibrosis, regions of fibrosis were not observed grossly nor was syncytial cell formation in bronchi or bronchioles a feature of the pneumonia in these donkeys.

**Table 1.** Diagnosis and ancillary tests performed on 13 feral donkeys (*Equus asinus*) after a mortality event.

Animal ID	Sex	Age (y)	Diagnoses	Pulmonary fibrosis	Bacterial culture*	PCR EHV1/EHV4, equine IAV†	Consensus herpesvirus PCRs‡
1	M	NA	Pneumonia, nephrosis, tracheitis	No	*Streptococcus equi* subsp. *zooepidemicus*	–	+
2	M	25	Pneumonia, emaciation, nematodiasis	Yes	NG	–	+
3	M	2	Pneumonia, hepatitis	Yes	NG	–	+
4	M	NA	Hemoabdomen, eosinophilic colitis	No	*Bacillus licheniformis*	–	–
5	F	4	Pneumonia, eosinophilic colitis, interstitial nephritis	Yes	NG	–	+
6	M	1	Pneumonia, eosinophilic colitis	Yes	NG	–	+
7	M	25	Pneumonia, eosinophilic enteritis	Yes	*Escherichia coli*	–	+
8	F	20	Pneumonia, eosinophilic enteritis	Yes	NG	NP	+
9	F	12	Pneumonia, eosinophilic enteritis	Yes	NG	NP	+
10	M	30	Pneumonia, eosinophilic enteritis, *Salmonella* Typhimurium enteritis	Yes	NG	NP	+
11	F	6	Pneumonia	Yes	*Escherichia coli*	NP	+
12	F	NA	Parasitism	No	NG	NP	–
13	F	7	Pneumonia, eosinophilic colitis, strangles (*Streptococcus equi* subsp. *equi*)	Yes	*Streptococcus equi* subsp. *zooepidemicus*	NP	+

EHV = equid alphaherpesvirus; F = female; IAV = influenza A virus; M = male; NA = not available; NG = no growth; NP = not performed; + = positive; – = negative.

* Culture was performed from lung tissues; additional culture from an abscessed medial retropharyngeal lymph node was performed in case C1043.

† PCR was performed from nasal swabs and lung tissues.

‡ Consensus herpesvirus PCR assays targeted the DNA polymerase gene and the DNA packaging terminase subunit 1 gene.

**Figures 1, 2.**
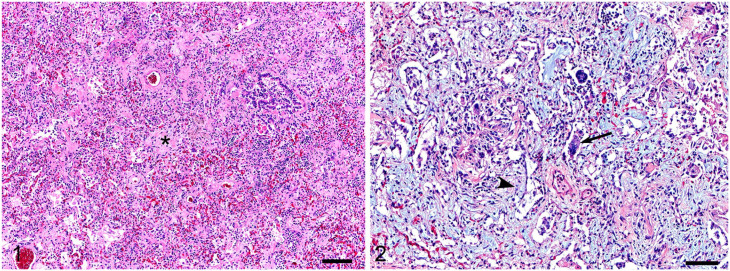
Histologic lesions of asinine gammaherpesviruses associated with pneumonia in donkeys. **Figure 1.** Interstitial fibrosing pneumonia in a donkey. In large sections of affected lungs, well-organized collagen bundles (asterisk) expand alveolar septa and compress adjacent alveoli. H&E. 100×. Bar = 400 μm. **Figure 2.** Interstitial fibrosing pneumonia with giant cells in a donkey lung. Abundant myxomatous stroma, loose collagen fibers, and rare lymphocytes and plasma cells expand alveolar septa and compress alveoli (arrowhead). Some alveoli contain cell debris, macrophages, and multinucleate cells (arrow). H&E. 100×. Bar = 200 µm.

Following initial presumptive diagnosis, equine influenza A virus and equid alphaherpesvirus 1 and 4 (EHV1, EHV4; subfamily *Alphaherpesvirinae*) were investigated by quantitative PCR.^5,14,16^ However, none of these viruses was detected in nasal swabs or lung tissues (*n* = 7; Table 1). Given that other members of the *Herpesviridae* subfamilies could be associated with the presented cases, consensus PCR assays directed towards the DNA polymerase gene^17^ and the DNA packaging terminase subunit 1 gene^6^ were performed on lung tissues (*n* = 13). Herpesviral DNA was detected in all animals from which fibrosing and pyogranulomatous pneumonia were initially diagnosed. Herpesviral DNA was not detected in animals without evidence of respiratory disease within the same herd (*n* = 2; Table 1).

In an attempt to classify the detected herpesvirus, partial gene sequencing was used to characterize related viral sequences. Consensus PCR products were purified (QIAquick PCR purification kit; Qiagen), and 6 that yielded sufficient quality were sequenced by the Sanger method at the Athens Veterinary Diagnostic Laboratory (Athens, GA, USA; SeqStudio, Thermo Scientific). Sequence identity was confirmed by BLAST (http://www.ncbi.nlm.nih.gov/blast/Blast.cgi). DNA packaging gene sequences (*n* = 3) were 98.8–99.7% identical to those from AsHV4 available in GenBank (Table 2). Similarly, DNA polymerase gene sequences (*n* = 4) were 97.2–100% identical to sequences from AsHV4 and AsHV5 (Table 2). Sequences generated in our study were deposited in GenBank (Table 2). Phylogenetic trees were generated using the amplified portion of the DNA polymerase and packaging terminase genes obtained by PCR assays. The phylogenetic analyses were inferred using MEGA X,^8^ with the maximum likelihood method based on the Tamura 3-parameter model.^15^ According to the packaging terminase-based phylogenetic tree, our sequences are grouped with AsHV4 strains; the presence of both AsHV4 and AsHV5 in this outbreak is indicated by the topology of the polymerase-based analysis (Suppl. Figs. 1, 2).

**Table 2.** Percentage of nucleotide sequence identity of DNA polymerase and packaging terminase genes with gammaherpesvirus sequences available in GenBank.

Animal ID	DNA polymerase gene		DNA packaging terminase gene	
	% identity	GenBank accession	% identity	GenBank accession
1	98.8 AsHV4	MW554696	99.74 AsHV4	MW554697
2	94.78 wild ass herpesvirus*	MT670426	NA	NA
3	100 AsHV5	MT670425	NA	NA
5	NA	NA	99.74 AsHV4	MW554698
8	NA	NA	99.74 AsHV4	MW554699
13	97.2 AsHV5	MT670428	NA	NA

AsHV = asinine herpesvirus; NA = not available, or sequencing was not performed. % identity = percentage of identity with AsHV sequences available in GenBank.

* Closely related to AsHV5 in the phylogenetic analysis (Suppl. Fig. 2).

Additional ancillary testing included aerobic culture from lung tissues over the course of the respiratory disease outbreak using 5% sheep blood and MacConkey agars, which were positive for *Streptococcus equi* subsp. *zooepidemicus* (*n* = 2), *Escherichia coli* (*n* = 2), and *Bacillus licheniformis* (*n* = 1, very light growth; Table 1). *S. equi* subsp. *equi* (*n* = 1) was isolated from an abscessed medial retropharyngeal lymph node of a donkey with strangles. Additional microscopic findings included mild eosinophilic enteritis and colitis (*n* = 8; Table 1); therefore, fecal egg counts (McMaster egg-counting technique) and aerobic bacterial culture of fecal samples were performed. Numerous strongyle-type eggs were seen in colon content from 3 animals (100–825 eggs/g), and *Salmonella enterica* subsp. *enterica* serovar Typhimurium was isolated from 1 animal (Table 1). In addition, a 29-trace element screen (mineral analysis) was performed on 6 liver samples. No significant mineral deficiencies or excesses at toxic concentration were detected.

We detected AsHV4 and AsHV5 consistently in lung tissues characterized by fibrosing pneumonia after a mortality event of recently captured feral donkeys. No other herpesviruses were identified, and herpesviral DNA was not detected in lung tissues without evidence of respiratory disease, suggesting that the detection of asinine gammaherpesviruses was associated with the described lesions. Similar findings were described in 11 donkeys with pulmonary lesions positive for AsHV4 (5 cases) and AsHV5 (6 cases), and in 6 asymptomatic donkeys that had no pulmonary lesions and were negative for asinine gammaherpesvirus DNA.^7^ In our study, a single animal had pyogranulomatous pneumonia, which was positive for AsHV4. Pyogranulomatous pneumonia attributable to AsHV5 has been described in a mare with persistent respiratory disease.^4^

We can only say that asinine gammaherpesviruses were associated with respiratory disease because it is conceivable that other factors and etiologic agents might have been involved in the initiation and progression of the lesions. As postulated,^3^ it is likely that pulmonary fibrosis in this disease outbreak was multifactorial involving both gammaherpesviral infection and primary pulmonary injury. In addition, gammaherpesviral infection could be linked to pulmonary fibrosis because these viruses modulate immunity by stimulating T helper-2 (Th2) rather than Th1 cell response, and fibrosis is more likely to develop within inflammation driven by Th2 cells.^19^ Given that herpesviruses are characterized by latency, with reactivation and shedding at times of stress and debilitation, it is likely that the virus was present in the feral herd with several persistently infected animals at the time of the roundup. The stress of capture along with concurrent disease such as parasitism, bacterial pneumonia in some cases, or exposure to pulmonary irritants such as dust or ammonia, may have caused reactivation of the virus, thus contributing to the development of pulmonary fibrosis. Unfortunately, given the remote location and timing, the 13 other donkeys with fatal outcome were not submitted for autopsy. No further epidemiologic data was available to investigate specific links among the affected individuals or to make comparisons with their peers.

Evolutionary analysis showed that AsHV4 and AsHV5 appeared to be distinct from each other, and most similar to EHV2 and EHV5. Similarly, asinine gammaherpesviruses have been shown to be closely related to EHV2 and EHV5.^6,13^ To date, isolation of AsHV4 and AsHV5 has been unsuccessful,^6,13^ with the majority of studies based solely on short gene sequences (< 800 bp) available in GenBank. Therefore, our results should be interpreted cautiously in determining whether the generated sequences belong to AsHV4 and 5 or possibly to other unclassified equid gammaherpesviruses.

Gammaherpesviruses have also been associated with pulmonary fibrosis in other animal species, such as horses, humans, and mice. However, the pathogenesis of such diseases is unclear in all animal species. EHV5 has been implicated in equine multinodular pulmonary fibrosis,^19,20^ with experimental induction of this condition in horses using EHV5 clinical isolates^21^; however, the choice of viral strain, immunologic status of experimental animals, and inoculation route may have favored the outcome of that experiment. Similarly, infection with gammaherpesviruses, particularly the Epstein-Barr virus, has been credited to an increased risk of pulmonary fibrosis development in humans.^19^ Further evidence for gammaherpesviral involvement in the development of pulmonary fibrosis has been provided by studies of lung infection in mice using murid gammaherpesvirus 68, an isolate of murid gammaherpesvirus 4 (genus *Rhadinovirus*).^19^ In dogs and cats, idiopathic pulmonary fibrosis shares some features with the human condition, however, no evidence of herpesviral infection associated with canine and feline pulmonary fibrosis has been reported.^12,19^

Findings in our case series suggest that gammaherpesviruses may be prevalent in this feral equid population and may contribute to post-gathering clinical disease. Whereas the detection of these viruses, here and in previous studies, does not prove disease causation, its potential association with respiratory conditions in equids deserves further attention. Our study contributes to public databases by providing novel nucleotide gene sequences of asinine gammaherpesviruses, for which only limited data currently exist. Further work is necessary to investigate the prevalence of asinine gammaherpesviruses within donkey populations and to elucidate their role in the development of pulmonary disease.

## Supplemental Material

sj-pdf-1-vdi-10.1177_10406387211052998 – Supplemental material for Detection of asinine gammaherpesviruses in association with pulmonary fibrosis in free-ranging donkeys Click here for additional data file. Supplemental material, sj-pdf-1-vdi-10.1177_10406387211052998 for Detection of asinine gammaherpesviruses in association with pulmonary fibrosis in free-ranging donkeys by Grazieli Maboni, E. Jane Kelly, Chad S. Clancy, Eliana De Luca, Thomas J. Baldwin, Arnaud J. Van Wettere, Albert J. Kane, Summer Peterson, V. Gus Warr, Dona A. Bastian and Susan Sanchez in Journal of Veterinary Diagnostic Investigation
