# Supplementary material for: Detection of asinine gammaherpesviruses in association with pulmonary fibrosis in free-ranging donkeys
Source: J Vet Diagn Invest. 2021 Oct 23;34(1):167–71. doi: 10.1177/10406387211052998 (PMC8688973; doi:10.1177/10406387211052998)
Supplement: sj-pdf-1-vdi-10.1177_10406387211052998 – Supplemental material for Detection of asinine gammaherpesviruses in association with pulmonary fibrosis in free-ranging donkeys [file sj-pdf-1-vdi-10.1177_10406387211052998.pdf]

JVDI: Supplemental material

Maboni G, et al. Detection of asinine gammaherpesviruses in association with pulmonary fibrosis in free-ranging donkeys

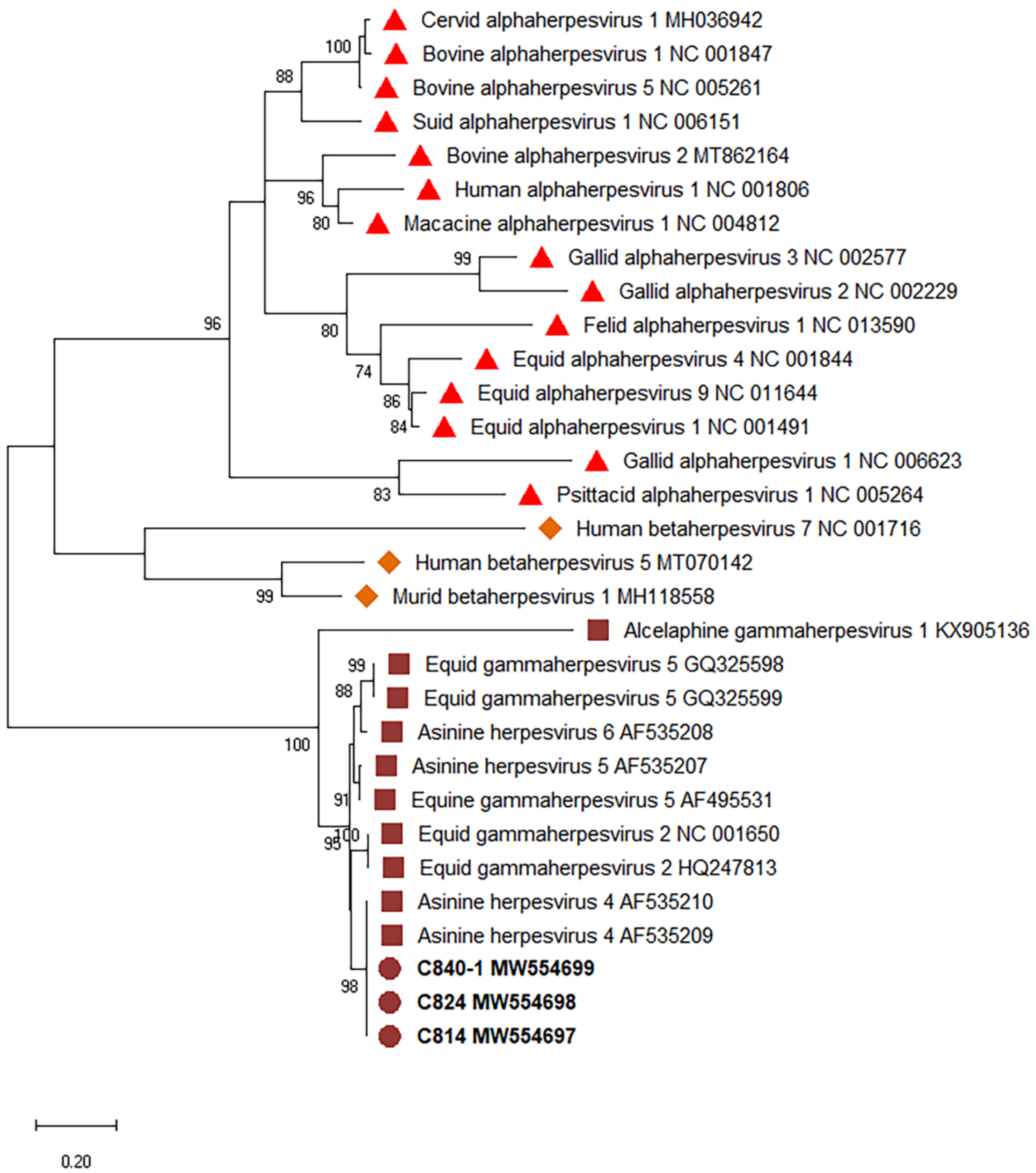

Supplemental Figure 1. Phylogenetic analysis based on the partial DNA packaging terminase gene nucleotide sequences of 3 sequences from our study and 21 representative strains

of the *Herpesviridae* family. The evolutionary history was inferred by using the maximum likelihood method and Tamura 3-parameter model. The percentage of trees in which the associated taxa clustered is shown next to the branches. A discrete gamma distribution was used to model evolutionary rate differences among sites (5 categories [+G, parameter = 0.7507]). The rate variation model allowed for some sites to be evolutionarily invariable ([+I], 0.00% sites). The tree is drawn to scale, with branch lengths measured in the number of substitutions per site. GenBank accessions are indicated in each branch. The sequences from our study (brown circle) are in bold. *Alphaherpesvirinae* subfamily (red triangle); *Betaherpesvirinae* subfamily (orange rhombus); *Gammaherpesvirinae* subfamily (brown square).

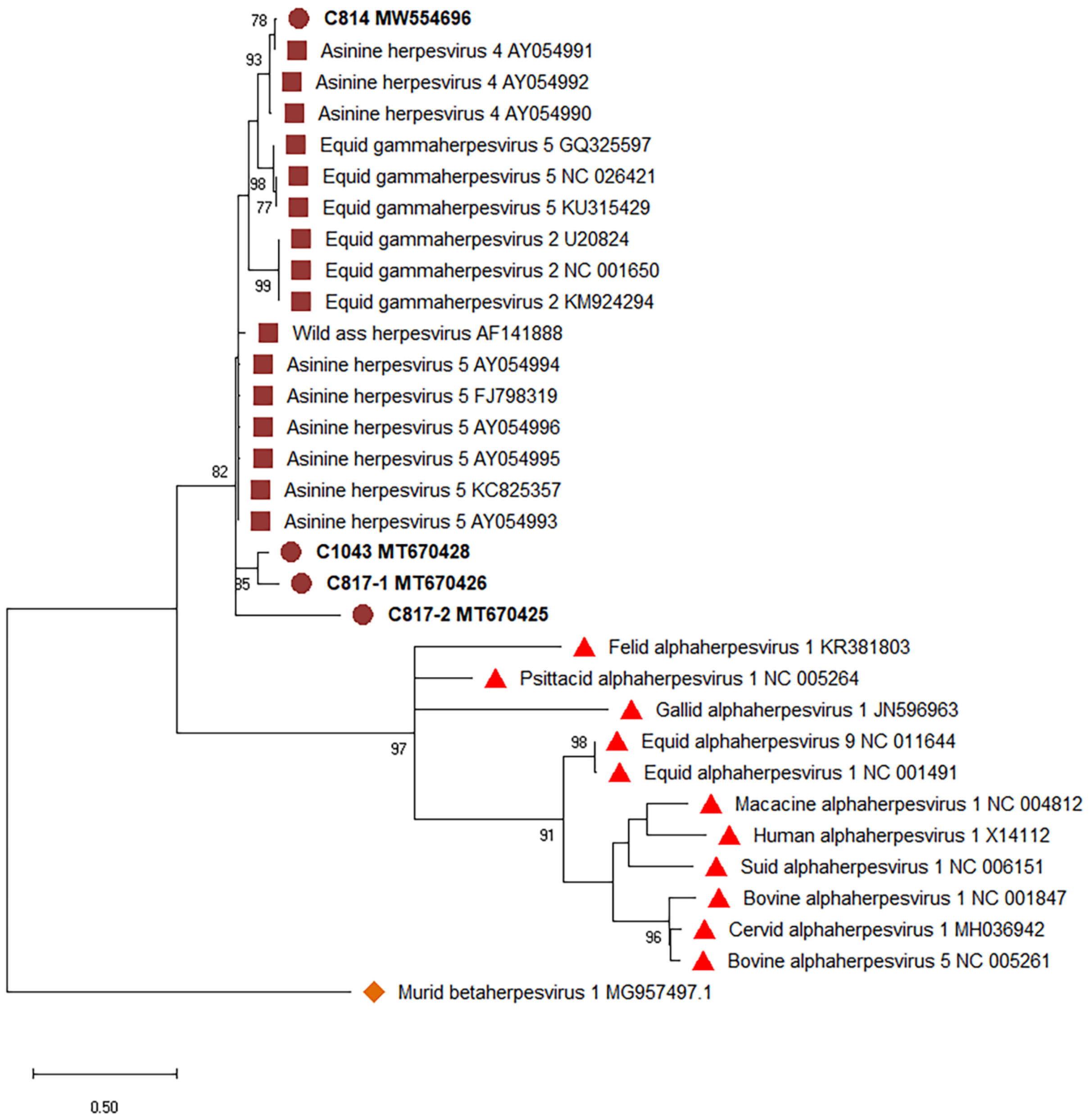

Supplemental Figure 2. Phylogenetic analysis based on the partial DNA polymerase nucleotide sequences of 4 sequences from our study and 28 representative strains of the *Herpesviridae* family. The evolutionary history was inferred by using the maximum likelihood method and Tamura 3-parameter model. The percentage of trees in which the associated taxa clustered is shown next to the branches. A discrete gamma distribution was used to model evolutionary rate differences among sites (5 categories [+G, parameter = 1.0222]). The rate

variation model allowed for some sites to be evolutionarily invariable ([+I], 0.00% sites). The tree is drawn to scale, with branch lengths measured in the number of substitutions per site. GenBank accessions are indicated in each branch. The sequences from our study (brown circle) are in bold. *Alphaherpesvirinae* subfamily (red triangle); *Betaherpesvirinae* subfamily (orange rhombus); *Gammaherpesvirinae* subfamily (brown square).
